# Complete genome organization of American hop latent virus and its relationship to carlaviruses

**DOI:** 10.1007/s00705-012-1312-0

**Published:** 2012-04-17

**Authors:** Kenneth C. Eastwell, Keri L. Druffel

**Affiliations:** 1 Department of Plant Pathology, Washington State University, IAREC, 24106 North Bunn Road, Prosser, WA 99350 USA; 2 Department of Plant Pathology, Washington State University, P.O. Box 646430, Pullman, WA 99164-6430 USA

## Abstract

The complete genomic sequence of American hop latent virus (AHLV; genus *Carlavirus*) was determined. The genome consists of 8,601 nucleotides plus a 3′-polyadenylate tail. The genome encompasses six potential open reading frames (ORF) in the positive sense, and their organization is typical of other carlaviruses. Analysis of the coat protein coding sequence at both the nucleic acid level and the amino acid level indicates that AHLV is only remotely related to the other carlaviruses known to infect common hop. Polyclonal antibodies were produced against the bacterially expressed coat protein of AHLV. These antibodies differentiated between AHLV and other carlaviruses of hop.

American hop latent virus (AHLV) (order *Tymovirales*, family *Betaflexiviridae*, genus *Carlavirus*) [1] is one of three carlaviruses that infect hop plants (*Humulus lupulus* L.). Mixed infections in hop of AHLV with other carlaviruses hop mosaic virus (HpMV) and hop latent virus (HpLV) are common [8], even in plants that do not express overt symptoms. All three of the carlaviruses known to infect hop are transmitted in a non-persistent manner by the damson hop aphid, *Myzus* (*Phorodon*) *humuli*, and it is presumed that this is the major route of natural transmission in the field [2]. Although natural spread in commercial hop plantings can be quite rapid, experimental aphid transmission of these carlaviruses is inefficient. In spite of the shared insect vectors, surveys of hop plantings indicate that AHLV generally occurs at a lower frequency than the other carlaviruses [8].

AHLV was first detected in commercial hops growing in North America [10] in plants that were also infected with other carlaviruses; AHLV was subsequently differentiated and further characterized in Europe in 1982 [2]. A comparative study examined the effects of HpMV, HpLV and AHLV on the cultivar Chinook [9]. AHLV reduced cone yield by 14 %, resulting in a reduction in the total yield of alpha acids by 12 %. A significant but slight impact was recorded on other agronomic traits such as plant height and lateral length. In general, the consequences of infection by carlaviruses on hop cone brewing characteristics appear to mimic changes associated with advanced maturity. The objective of the study reported herein is to develop a greater knowledge of the viruses infecting hop plants.

Initial cloning and sequencing of AHLV was performed from partially purified virus from *Chenopodium quinoa* mechanically inoculated with a virus isolate from *H. lupulus* ‘Galena’. After a preliminary genome sequence was established for the virus maintained in *C. quinoa* (GenBank JQ728538), the complete genomic sequence of AHLV was derived directly from *H. lupulus* ‘Bittergold’. AHLV was enriched from hop leaf extracts by treatment with bentonite followed by polyethylene glycol precipitation [4]. Viral RNA was precipitated after proteinase K digestion of the partially purified virions [11]. The complete genome was cloned and sequenced using a combination of cDNA cloning and 5′-RACE (Invitrogen, Carlsbad, CA) as described previously [5], and the sequence was deposited in the public database (GenBank JQ245696).

The complete coat protein coding sequence was amplified from ‘Bittergold’ using primers AHLVcpF: 5′-CCTTTTTTTTTCCCATATGTCGAACGTTGAAAGG-3′and AHLVcpR: 5′-TTATGGATCCTCAGTGCGCTTGTCGAAACTC-3′. The primers were extended (underlined sequence) to allow digestion of the amplified product with *Nde*I and *Bam*HI and it was then ligated into plasmid pET15b (Novagen: San Diego, CA). *E. coli* Rosetta™ competent cells (Novagen) were transformed with the plasmids, and proteins were expressed following the manufacturer’s recommendations. Fusion proteins produced with the N-terminal His-tag were isolated and concentrated using a Ni column (ProBond™ Resin; Invitrogen) according to manufacturer’s instructions. New Zealand white rabbits were immunized with four injections of 400 μg each of the resulting protein, and antibodies were evaluated by indirect ELISA with absorbance values determined two hours after substrate addition.

The complete genome of AHLV from ‘Bittergold’ consists of 8,601 nucleotides (nt) plus a poly-adenylated tract at the 3′-terminus. The genome contains six potential open reading frames (ORFs) organized in a pattern typical of members of the genus *Carlavirus*. ORF1 spans nt positions 82 to 6015 and encodes a protein of 1,977 amino acids (aa) with a predicted molecular mass of 224.4 kDa. This corresponds to the largest protein produced through *in vitro* translation of isolated virus RNA [6]. The predicted protein contains domains characteristic of virus replicases, including a viral methyl-transferase domain (aa residues 43 to 359) involved in mRNA capping. A 5′ cap structure was not detected by the methods used in this study but is inferred by the similarity of AHLV to other members of the genus *Carlavirus* and by *in vitro* translation data [6]. Other signature conserved domains include the RNA-dependent polymerase family domain (aa residues 1589 to 1967), a carlavirus-specific endopeptidase (aa residues 998 to 1086), and a viral RNA helicase (aa 1172 to 1300). The replicase polyfunctional protein of AHLV shows 36 to 42 % identity to nt sequences and 35 to 48 % identity to aa sequences of the known carlaviruses (Fig. 1A). This is consistent with the position of AHLV as a member of a distinct species within the genus *Calarvirus* [1].

**Fig. 1:**
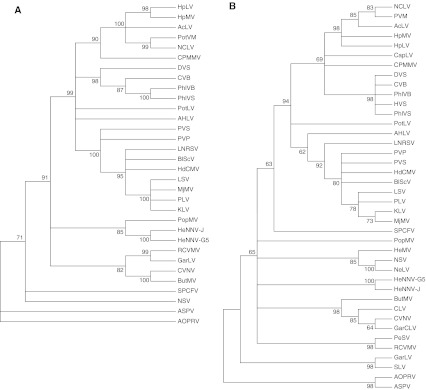
Cladogram of the predicted amino acid sequences from the replicase (**A**), and coat protein (**B**) of members of the genus *Carlavirus*. The phylogenetic tree was generated using the maximum-likelihood method in the MEGA5 analysis package. Branch significance was evaluated by constructing 1,000 trees in bootstrap analysis, and the bootstrap values (>60 %) are shown. The sequences used were derived from the genomic sequences of Aconitum latent virus (AcLV, gi: 14251190), American hop latent virus (AHLV, gi: JQ245696), blueberry scorch virus (BlScV, gi: 19919919), butterbur mosaic virus (ButMV, gi: 269954638), chrysanthemum virus B (CVB, gi: 154814487), coleus vein necrosis virus (CVNV, gi: 156616260), cowpea mild mottle virus (CPMMV, gi: 313122763), daphne virus S (DVS, gi: 94971809), garlic latent virus (GarLV, gi: 20143432), Helleborus net necrosis virus (HeNNV-G5, gi: 222509601 and HeNNV-J, gi: 349584640), hop latent virus (HpLV, gi: 10314012), hop mosaic virus (HpMV, gi: 171473627), hydrangea chlorotic mottle virus (HdCMV, gi: 242346753), Kalanchoe latent virus (KLV, gi: 254667440), Ligustrum necrotic ringspot virus (LNRSV, gi: 166851938), lily symptomless virus (LSV, gi: 37674199), Mirabilis jalapa mottle virus (MjMV, gi: 352749693), narcissus common latent virus (NCLV, gi: 110835685), narcissus symptomless virus (NSV, gi: 116734779), Passiflora latent virus (PLV, gi: 113195533), phlox virus B (PhlVB, gi: 160700642), phlox virus S (PhlVS, gi: 145651764), poplar mosaic virus (PopMV, gi:4 1057345), potato latent virus (PLV, gi: 211998869), potato virus M (PVM, gi: 72257063), potato virus P (PVP, gi: 156603882), potato virus S (PVS, gi: 71849435), red clover vein mosaic virus (RCVMV, gi: 224923880), and sweet potato chlorotic fleck virus (SPCFV, gi: 56692800). In addition, the following carlaviruses were included in the analysis of coat protein sequences: caper latent virus (CapLV, gi: 333034471), cole latent virus (CLV, gi: 33517421), garlic common latent virus (GarCLV, gi: 3077682), Helenium virus S (HVS, gi: 221446), Helleborus mosaic virus (HeMV, gi: 222142735), Nerine latent virus (NeLV, gi: 298504072), pea streak virus (PeSV, gi: 13811416), shallot latent virus (SLV, gi: 3077693). The corresponding sequences of apple stem pitting virus (ASPV, gi: 19744938) and African oil palm ringspot virus (AOPRV, gi: 226202432) were used as out-groups

Overlapping ORFs 2, 3 and 4 span nt positions 6068 to 6772, 6741 to 7061 and 7034 to 7234, respectively, and encode proteins of 234 aa (26.2 kDa), 106 aa (11.3 kDa) and 66 aa (7.3 kDa) with homology to the triple gene block proteins of virus superfamily 1.

ORF5 of the AHLV genome encodes a capsid protein with a molecular mass of 36.0 kDa based on the predicted aa sequence. This mass is consistent with a protein produced in earlier wheat germ *in vitro* translation experiments and precipitated by virion-specific antibodies [6]. The molecular mass of the AHLV coat protein is within the range of 31–36 kDa anticipated for members of the genus *Carlavirus* and contains two highly conserved domains typical of carlaviruses and potexviruses. Antibodies produced to the bacterially-expressed coat protein of AHLV reacted preferentially with AHLV-infected hop leaf extracts (3.6 times above non-infected hop tissue), while there was no significant reaction to HpLV or HpMV (1.2 times above non-infected hop samples). This is similar to serological data produced with antibodies against purified virions [3]. Whereas HpLV and HpMV share distant antigenic similarities with each other and with many other carlaviruses, AHLV is only remotely serologically related to other carlaviruses, if at all [3]. This distinction is reflected by phylogenetic analysis of the coat protein sequences from carlaviruses (Fig. 1B). The predicted aa sequences of the coat proteins of HpMV and HpLV share 66 % identity with each other, but only 37 % and 38 % identity to AHLV, respectively. Moreover, the coat protein of AHLV contains 323 aa, while those of HpMV and HpLV contain 307 and 306 aa, respectively. When compared to the coat proteins of other carlaviruses, the coat protein of AHLV shows 14 to 47 % nt sequence identity and 26 to 49 % aa identity.

There is no current information regarding possible strains of AHLV. However, the two AHLV isolates determined in this study share overall sequence identity of 98 % at the nt level. The coat protein coding sequences share 99 % nt identity and 98 % aa identity.

ORF6 of AHLV encodes a 114-aa protein (13.1 kDa) with homology to the carlavirus putative nucleic-acid-binding protein, including the highly conserved cysteine residue motif, consistent with the presence of a nucleic-acid-binding zinc finger [7].

These data clearly demonstrate the position of AHLV as a member of a unique species of the genus *Carlavirus* [1] and reflect the biological data that suggest that it is distantly related to the other carlaviruses associated with hop plants. This study provides the foundation of further investigation into the differentiation of AHLV into strains and their role in the pathology of disease associated with hop.
